# *In vitro* comparison of tapered versus standard bristle toothbrushes for plaque removal and peri-implant disease prevention

**DOI:** 10.6026/973206300222154

**Published:** 2026-04-30

**Authors:** Abhishek Sinha, Tank Samprada, Pavithra Rangarajan Seshadri, Shalaka Raurale, Rajeev Shrivastava, Arvind Jain, Miral Mehta

**Affiliations:** 1 Department of Periodontology, Purvanchal Institute of Dental Sciences, Gorakhpur, Uttar Pradesh, India; 2 Department of Pharmacology, Smt. B. K. Shah Medical Institute & Research Centre, Sumandeep Vidyapeeth (Deemed to be University), Vadodara, Gujarat, India; 3 Department of Periodontics, Ragas Dental College and Hospital, Uthandi, Chennai, Tamil Nadu, India; 4 Department of Orthodontics and Dentofacial Orthopaedics, Dr. N. Y. Tasgaonkar Institute of Medical Science and Research Center, Diksal, Karjat, Maharashtra, India; 5 Department of Prosthodontics and Crown & Bridge, New Horizon Dental College and Research Institute, Bilaspur, Chhattishgarh, India; 6 Department of Oral Pathology and Microbiology, Maitri College of Dentistry and Research Centre Durg, Chhattisgarh, India; 7 Department of Pediatric and Preventive Dentistry, Karnavati School of Dentistry, Karnavati University, Gandhinagar, Gujarat, India

**Keywords:** Tapered bristles, dental implant, plaque removal, peri-implant disease, toothbrush, biofilm, surface roughness

## Abstract

Peri-implant diseases pose significant clinical challenges in implant dentistry, with inadequate plaque control identified as a primary risk factor, yet the effects of bristle geometry on peri-implant plaque removal remain poorly understood. Therefore, it is of interest to compare tapered and standard flat-cut bristle toothbrushes under manual and powered brushing conditions on 40 implant-abutment models with simulated soft tissue. Plaque removal, subgingival bristle penetration, surface cleaning and titanium surface integrity were assessed via digital planimetry, micro-CT, SEM and profilometry. Tapered bristles demonstrated superior plaque removal in sulcular/interproximal areas and deeper subgingival penetration with minimal surface roughness versus standard bristles. Thus, tapered-bristle toothbrushes are the optimal choice for peri-implant plaque control, thereby reducing disease risk while preserving abutment integrity.

## Background:

Dental implants have become the current standard of care for replacing missing teeth and 95-year survival rates have been reported to be high in many longitudinal studies [1]. The biological complications of dental implants, especially peri-implant mucositis and peri-implantitis, have become a serious threat to implant survival and one of the most topical issues in modern implant dentistry [2]. According to the epidemiological data, peri-implant mucositis occurs in about 43 to 80% of patients with implants and peri-implantitis, which is manifested by the progressive marginal bone loss around the implants that are osseointegrated, has a prevalence of between 12 and 43 percent, based on the diagnostic criteria and the population under study [3]. The etiology of peri-implant diseases shares commonalities with periodontal diseases, with bacterial biofilm deposits on implant and abutment surfaces playing the primary initiating role [4]. Lack of periodontal ligament fibers and disrupted soft tissue structure around implants predispose the peri-implant environment to bacterial colonization and inflammatory degradation relative to the natural dentogingival complex [5]. The supracrestal attachment of connective tissue to the implants, sometimes called a biological seal, is structurally weak and less resistant to bacterial entry than the junctional epithelium and connective tissue attachment to the apical parts of natural teeth [6]. Therefore, careful and regular mechanical plaque control around dental implants is considered key to preserving peri-implant tissue and preventing reversible mucositis from progressing to irreversible peri-implantitis [7]. Brushing of the teeth is the basis of daily oral care. It is also the most widely used form of biofilm management, whether in natural teeth or on implant-supported restorations [8]. Toothbrush design, such as bristle material, diameter, tip design, stiffness, arrangement pattern and head geometry, also significantly impacts the cleaning effectiveness of brushes and their compatibility with the tissue [9]. The bristle-tip design is one such variable that has gained increasing attention as a means to improve plaque removal in anatomically difficult regions with minimal soft-tissue trauma [10].

Traditional toothbrushes use flat or end-rounded bristle filaments of uniform diameter, commonly 0.15 to 0.25 mm, that provide uniform cleaning action throughout the contact zone of the bristle tip [11]. Conversely, the tapered bristle toothbrushes have filaments that are either mechanically or chemically treated to create a steady decrease in diameter from the base to the end, yielding ultrafine bristle endings with diameters as small as 0.01 mm [12]. This tapered shape ideally enables the thin ends of the bristles to reach deeper into the gingival sulcus, spaces between teeth and other parts of the intricate morphology that cannot be reached with normal diameter bristle tips [13]. Various clinical trials have shown the potential benefits of tapered-bristle toothbrushes for the elimination of subgingival plaque and the reduction of gingival inflammation in the area of natural teeth [14]. Investigations have demonstrated that tapered bristles penetrate periodontal pockets more deeply than flat-cut ones and penetration depths of up to 1.5 mm below the gingival margin have been reported under controlled brushing conditions using tapered bristles [15]. Although the findings are promising for maintaining peri-implant periodontal health, the implications for peri-implant plaque control have received very little research. The peri-implant situation offers a contrasting environment to the natural periodontal setting due to its peculiarities. The emergence mode of implant-supported restorations tends to form concavities and irregularities that entrap biofilm; the titanium abutment surface topography is not similar to that of the physiological tooth; and the peri-implant sulcus depth is often larger than the physiological sulcus around natural teeth [16]. Moreover, the vulnerability of titanium surfaces to mechanical stresses generated by oral hygiene instruments raises questions about whether contact with toothbrush bristles can induce surface changes that enable bacterial adhesion and compromise the biocompatibility of the implant-abutment interface [17]. There is a major gap in the literature on the comparative performance of various bristle configurations in specific peri-implant applications. The bulk of the existing evidence on the design of tooth bristles has been developed using natural tooth models and the direct transfer of these results to implant conditions may be invalid due to anatomical, material and microbiological differences in the peri-implant environment [18]. Therefore, it is of interest to compare the effectiveness of tapered bristle toothbrushes with flat-cut bristle toothbrushes in the removal of the plaque around the dental implant abutments, the depth of subgingival bristle penetration and repeated brushing on the titanium abutment surface integrity under standardized experimental conditions.

## Materials and Methods:

## Study design:

This was a controlled *in vitro* experimental research study carried out at the Department of Periodontology and Implant Dentistry in collaboration with the Biomaterials Research Laboratory.

## Fabrication of implant-Abutment assembly and model:

They were 40 titanium dental implants (diameter: 4.1 mm, length: 10 mm, machined collar with moderately rough body) and 40 straight titanium abutments (diameter: 4.5 mm, gingival height: 3 mm, collar height: 1 mm) of one manufacturer (Straumann AG, Basel, Switzerland). The implants were placed vertically in auto-polymerizing acrylic resin in a standardized typodont base that represents the posterior mandibular region and the implant platforms were placed 2 mm below the simulated bone crest levels to simulate a protocol of subcrestal positions. Implants were attached to abutments and tightened to 35 Ncm, per the manufacturer's recommendations, using a calibrated torque wrench. An abutment assembly was surrounded by simulated peri-implant soft tissue, a tested silicone-based gingival analog material (GI-Mask Automix, Coltene, Altstätten, Switzerland), which was moulded around the abutment assembly to form a standardized peri-implant sulcus depth of 3 mm circumferentially. The silicone compound was chosen for its tissue-like nature and its ability to replicate the resistance and displacement properties of peri-implant mucosa when using a toothbrush bristle. All assemblies were placed in the typodont to replicate a single-tooth implant restoration at the first molar location, with the other acrylic teeth positioned next to the simulated interproximal contacts.

## The artificial biofilm application:

An artificial biofilm was created to be of standard type, simulating plaque in all specimens in a reproducible manner. A simulated plaque based on the artificial plaque, which was developed with a validated protocol in the simulation of plaque *in vitro* works, was made using the following components: hydroxyethyl cellulose (2%), carboxymethyl cellulose (1%), glycerine (5%), titanium dioxide (0.5% to provide opaceness) and fluorescent dye (fluorescein sodium, 0.1%). The abutment assemblies were placed in the artificial biofilm solution, immersed for 60 s, removed and left to dry at room temperature (23 ± 1°C) and 50 percent relative humidity. This was repeated three times to obtain a uniform, multi-layered biofilm coating of about 50-80 um thickness, as confirmed using a digital micrometer on the samples. The fluorescent component was used to quantitatively perform a digital planimetric analysis of the residual plaque after brushing.

## Group distribution and choice of toothbrush:

The forty assemblies of specimens were randomly assigned to four groups of ten specimens each by using a computer-generated randomization sequence:

[1] Group A (n = 10): Tapered bristle manual toothbrush.

[2] Group B (n = 10): Standard flat-cut bristle manual toothbrush

[3] Group C (n = 10): Tapered bristle powered toothbrush head.

[4] Group D (n = 10): normal flat-cut bristle-powered toothbrush head.

In the case of manual-brushing groups, commercially available soft-bristle toothbrushes from the same manufacturer were chosen to manipulate the head size and the arrangement of the bristles, varying only in the arrangement of the bristle tips. The tapered bristle toothbrush (Curaprox CS 5460 Ultra Soft, Curaden AG, Kriens, Switzerland) was provided with chemically tapered filaments with tips around 0.01 mm at the terminal end. The conventional end-rounded filaments had a diameter of, say, 0.20 mm and were in the standard bristle toothbrush (Oral-B Indicator 35 Soft, Procter and Gamble and Cincinnati, OH, USA). In the case of powered brushing groups, powered toothbrush bodies (Oral-B Pro 2000, Procter & Gamble) were used and powered toothbrush heads prepared: a tapered-tip replacement head (Oral-B Sensi UltraThin, Procter & Gamble) and an ordinary replacement head (Oral-B CrossAction, Procter and Gamble), both of which were driven by one powered toothbrush body at the standard cleaning mode setting.

## Learning brushing standard:

All brushing was performed using a specially designed brushing simulation device that provided control over the applied force, brushing angulation, stroke length and stroke duration. The equipment comprised a pneumatic actuator arm with an adjustable force transducer, a specimen mounting platform with angular adjustment and a digital controller for stroke parameters. The parameters of brushing were standardized as follows: (2 N ±0.1 N) constantly applied force, using a load cell; 45 degree relative to the long axis of abutment (Bass technique orientation); 15 mm stroke length; 4 Hz brushing rate in case of manual simulation and manufacturer-specified oscillation rate in case of electrical simulation; 30 seconds brushing time on each surface of the specimen (buccal, lingual, mesial, distal), which was summed to 2 minutes per specimen. The toothpaste used was standard slurry fluoride toothpaste and its weight was divided by 3 to obtain the deionized water-to-toothpaste ratio (1:3).

## Outcome assessments:

## Plaque removal efficacy:

Depaler photos were taken under ultraviolet light (wavelength of 405 nm) using a standardized imaging system in which a digital camera (Canon EOS 90D, equipped with a 100 mm macro lens) was placed on a fixed stand at a fixed distance (15 cm) with exposure settings set to standard (ISO 400, f/16 and 1/125 s). Four standardized angles of the fluorescent residual biofilm (buccal, lingual, mesial and distal) were taken on each specimen. ImageJ software (version 1.53, National Institutes of Health, Bethesda, MD, USA) was used to measure the percentage of remaining plaque on the abutment and peri-implant surfaces using the digital planimetric analysis. Regional analysis was done by splitting the total abutment surface into three areas: Zone 1 (supragingival abutment surface), Zone 2 (sulcular/marginal zone, between the gingival margin and 1 mm subgingival) and Zone 3 (interproximal zone, comprising mesial and distal abutment surfaces). The calculation of the percentage of plaque removal was done as: [(Pre-brushing plaque area- Post-brushing plaque area)/ Pre-brushing plaque area) x 100].

## Sub gingiva bristle penetration test:

Another group of five different specimens (not part of the main analysis of results) was prepared specifically for the bristle penetration test. A thin layer of radiopaque barium sulfate suspension was applied to the toothbrush bristles before brushing to visualize the maximum pathway of bristle penetration. Active brushing simulation: In active brushing simulation, the specimen was quickly clamped at the peak of maximum deflection using a fast-setting cyanoacrylate adhesive, which held the bristles in the deepest penetration position. A micro-computed tomography system (SkyScan 1275, Bruker, Kontich, Belgium) was used to scan these frozen assemblies at a resolution of 15,000 voxels, with the following parameters: 70 kVp, 166 µA and a 0.5 mm aluminum filter. NRecon software (Bruker) was used to create three-dimensional reconstructions and bristle penetration as measured at four points (buccal, lingual, mesial and distal) around the simulated gingival margin using CTAn analysis software (Bruker).

## Surface integrity evaluation:

To assess the impact of repeated contact between toothbrushes and titanium abutment surfaces, a different group of twenty abutments (five per group) was exposed to an accelerated wear regime involving 10,000 brushing strokes, with the same set of standardized force and angulation parameters, which would approximate six months of daily brushing. The arithmetic mean roughness (Ra) and maximum height roughness (Rz) over a measurement length of 4 mm at three standardized locations on each abutment were measured on surfaces without accelerated brushing protocol using a contact profilometer (Surftest SJ-410, Mitutoyo Corporation, Kawasaki, Japan) with a diamond stylus (tip radius 2 µm) before and after the accelerated brushing protocol. Also, a few specimens from both groups were examined using scanning electron microscopy (SEM) (JSM-6510LV, JEOL, Tokyo, Japan) at 500 x and 2000 x magnification to qualitatively assess surface morphology changes.

## Statistical analysis:

The SPSS software (version 27.0, IBM Corporation) was used to conduct all quantitative data analysis. The Shapiro-Wilk test was used to assess the normality of the data distribution. ANOVA was conducted in two ways, with the independent variables type of bristle (tapered or standard) and mode of brushing (manual or powered), to obtain results on plaque removal and bristle penetration. Inter-group comparisons were performed using one-way ANOVA with a post-hoc Tukey test. Intra-group pre- and post-brushing surface roughness was assessed using the paired t-tests. The significance level was set at p<0.05.

## Results:

All specimens completed the experimental protocols without damage or loss, yielding complete datasets for all forty primary specimens and the additional specimens designated for penetration and surface integrity analyses. Overall plaque removal percentages demonstrated significant main effects for both bristle type (p < 0.001) and brushing mode (p = 0.003), with no significant interaction effect (p = 0.284). Tapered-bristle toothbrushes achieved significantly higher overall plaque removal than standard-bristle counterparts, regardless of brushing mode. Regional analysis revealed that the most pronounced differences among bristle types were observed in the sulcular (Zone 2) and interproximal (Zone 3) regions. In contrast, differences in the supragingival zone (Zone 1) were less pronounced. Powered brushing groups outperformed manual groups across all zones and both bristle configurations (Table 1 - see PDF). Micro-CT analysis demonstrated significant differences in bristle penetration depth between tapered and standard bristle configurations at all measured circumferential positions. Tapered bristles achieved a mean overall penetration depth of 1.86 ± 0.42 mm below the simulated gingival margin, compared to 0.74 ± 0.28 mm for standard bristles in manual configurations and 2.14 ± 0.38 mm versus 0.92 ± 0.32 mm in powered configurations. The greatest penetration was observed at the buccal and lingual positions, while interproximal penetration was relatively reduced across all groups due to the spatial constraints imposed by adjacent teeth (Table 2 - see PDF). All groups demonstrated statistically significant increases in surface roughness parameters following the 10,000-stroke accelerated brushing protocol (p < 0.05 for all intra-group comparisons). However, the magnitude of roughness increase varied significantly among groups. Standard bristle groups produced significantly greater increases in both Ra and Rz values compared to tapered bristle groups (p < 0.001 for bristle type main effect). Powered brushing produced marginally greater roughness changes than manual brushing, though this difference reached statistical significance only for Ra in the standard bristle comparison. SEM evaluation confirmed these quantitative findings, revealing visible scratching patterns and surface abrasion marks that were more prominent and deeper in the standard bristle groups. In contrast, tapered bristle specimens showed minimal surface alterations with superficial, shallow marks (Table 3 - see PDF).

## Discussion:

The results of the current *in vitro* research provide strong evidence that tapered-bristle toothbrushes offer significant benefits over flat-cut-bristle toothbrushes in plaque removal within the peri-implant environment, especially in the clinically important sulcular and interproximal areas, where biofilm deposition poses the greatest risk to peri-implant tissue health. Moreover, the tapered bristles exhibit a more desirable profile for preserving titanium abutment surface integrity, indicating a two-fold benefit that supports their recommendation for maintaining the implants. The overall plaque removal efficiency observed with tapered bristles is also in line with results from clinical trials on natural teeth, where the effectiveness of fine brush tips in cleaning has been attributed to the accessibility of apex-isolated anatomical niches that standard-diameter filaments cannot reach [19]. The current research carried this evidence to the peri-implant setting and the benefit was the greatest in the sulcular area, with tapered bristles eliminating about 40 percent of the plaque compared to regular bristles in this area when brushing manually. This difference is clinically important because the peri-implant sulcus is the primary ecological niche for pathogenic biofilms, which promote the transition from peri-implant health to mucositis and, ultimately, to peri-implantitis [20]. The mechanistic basis of the observed differences in the removal of the sulcular plaque is the substantially greater subgingival penetration depth of tapered bristles, 1.86 mm on average in manual mode vs. 0.74 mm with standard bristles. A gradual reduction of the diameter of the bristles at the base, to a point of 0.01 mm at the tip, forms a flexible and highly compliant terminal section, which can manoeuvre easily through the tight constraint of the peri-implant sulcus without causing excessive lateral stress on the sulcular epithelium [21]. Past studies with natural tooth models had also found that the same penetration differentials were obtained, with tapered bristles always having an access of one to two millimeters deeper into the sulcus than end-rounded bristles [22]. The current data indicate that the above penetration advantage is maintained and perhaps, due to slightly broader and more uniform sulcus morphology about implant abutments than the variable sulcus form of the anatomy of natural teeth. The fact that powered brushing increased plaque removal in both bristle arrangements is consistent with extensive evidence supporting the greater efficacy of oscillating-rotating powered toothbrushes compared with manual brushing in total plaque removal [23]. Nevertheless, the additive effect of tapered bristles on powered brushing, which produced the highest plaque removal percentages across all areas, would suggest that bristle arrangement and brushing mechanism have independent and complementary roles in cleaning performance. The practical implications of this discovery can be applied to clinical recommendations, as patients with dental implants can achieve optimal benefits when using powered toothbrushes with tapered bristle heads [24]. A specific emphasis should be placed on data concerning interproximal plaque removal, since the interproximal area around the implant-supported restorations is one of the most problematic areas to address using biofilm control methods and is commonly the first site where peri-implant bone loss manifests [25]. Although it is commonly thought that brushing teeth alone is only a sufficient level of interproximal cleaning and complementary interdental hygiene aids are an excellent recommendation, the magnitude of interproximal plaque reduction provided by tapered bristles is significantly larger, implying that these types of brushes clean well in this area of the mouth, potentially minimizing the amount of biofilm remaining to be removed by adjunctive devices [26]. Surface roughness data are a crucial part of this study because, based on the literature on the biology of implants, the relationship between surface features and bacterial colonization has been well established. Ra = 0.2 µm has been suggested as the critical level, above which bacterial adhesion increases markedly and all post-brushing roughnesses in the current study exceed this value [27]. Nevertheless, the considerably smaller roughness gain with tapered bristles compared with standard bristles implies that the surface is kept closer to the desirable spectrum for a longer time, which could be extended until the surface deterioration changes to reach concentrations that substantially promote bacterial colonization [28]. The principle of reduced surface damage from the use of tapered bristles is associated with contact mechanics. The tapered bristle stub, with a very small contact area, produces less total force per bristle than the same total force used in brushing, since the flexible tapered region absorbs and shares the applied load over a longer length of deformation [29]. By contrast, the applied force on the blunt-end-rounded tip of standard bristles is transmitted more directly to the abutment surface and the stress is concentrated at the contact point, resulting in deeper abrasion marks, as SEM observations in this study verified. These findings have clinical implications not just in the immediate context of daily oral hygiene. Evidence should inform peri-implant maintenance protocols (which usually incorporate both professional and self-administered plaque-control measures) regarding the performance characteristics of the recommended hygiene instruments in the peri-implant setting [30]. The current findings indicate that the use of tapered- bristle toothbrushes in the peri-implant maintenance guidelines may contribute more effectively to biofilm prevention in both the critical sulcular and interproximal sites, whilst also averting the progressive mechanical loss of titanium abutment surfaces. This study has several limitations that should be acknowledged. The *in vitro* design, though it offers an opportunity to obtain standardized, reproducible experimental environments, cannot recreate the complexity of the intraoral environment, such as salivary flow, natural biofilm composition and maturation, variability of the brushing technique individual to patients, soft tissue resilience and the response of bleeding and the three-dimensionality of implant-supported restorations [31].

## Conclusion:

Tapered bristle toothbrushes significantly outperformed standard flat-cut bristles in peri-implant plaque removal, particularly in sulcular and interproximal areas critical for disease prevention. Their deeper subgingival penetration and reduced titanium surface roughness changes mechanistically justify superior biofilm disruption with minimal iatrogenic damage. Thus, tapered bristles with powered brushing emerge as the optimal recommendation for implant patients, warranting clinical validation through prospective randomized trials.
